# Does the Depth of Hysteroscopic Endometrial Fundal Incision Influence Reproductive Outcomes in Oocyte Recipients? A Prospective Study

**DOI:** 10.3390/jcm15124689

**Published:** 2026-06-17

**Authors:** Robert Najdecki, Nikolaos Peitsidis, Ioannis Tsakiridis, Evangelos Papanikolaou, Fotini Chouliara, Georgios Michos, Krzysztof Łuszczyński, Marcin Chlebus, Paweł Kamiński, Monika Szafarowska

**Affiliations:** 1 Assisting Nature Centre of Reproduction and Genetics, Thessaloniki GR-57001, Greece; docnajdecki@gmail.com (R.N.); nickpeits@windowslive.com (N.P.); iotsakir@gmail.com (I.T.); papanikolaou@assistingnature.gr (E.P.); chouliara@assistingnature.gr (F.C.); giomichos@hotmail.com (G.M.); 2 Third Department of Obstetrics and Gynaecology, School of Medicine, Faculty of Health Sciences, Aristotle University of Thessaloniki, Thessaloniki GR-54124, Greece; 3 Department of Histology and Embryology, Medical University of Warsaw, 02-004 Warsaw, Poland; kluszczynski@wim.mil.pl; 4 Department of Internal Medicine, Pneumonology, Allergology, Clinical Immunology and Rare Diseases, Military Institute of Medicine—National Research Institute, 04-141 Warsaw, Poland; 5 Faculty of Economic Sciences, University of Warsaw, 00-241 Warsaw, Poland; 6 Department of Gynecology and Oncological Gynecology, Military Institute of Medicine—National Research Institute, 128 Szaserów Street, 04-141 Warsaw, Poland; pkaminski@wim.mil.pl

**Keywords:** IVF, oocyte recipients, hysteroscopy, HEFI, pregnancy rate

## Abstract

**Background:** Hysteroscopic endometrial fundal incision (HEFI) is a novel addition to the standard IVF procedure. This study aimed to evaluate whether different depths of incision during HEFI could affect reproductive outcomes in oocyte recipients. **Methods:** A prospective analysis was conducted on women who underwent HEFI 1–2 months prior to embryo transfer with donor oocytes. Patients were categorized into three groups based on the depth of incision: U2a (superficial level) U2aa (intermediate level), and U2aaa (deep level). Pregnancy and live birth rates were assessed. **Results:** A total of 212 women without intrauterine pathology were included. Positive pregnancy rates were 78% in U2a, 76.9% in U2aa, and 77.1% in U2aaa (*p* = 0.95). Live birth rates were 58.5%, 57.1%, and 56.4%, respectively (*p* = 0.97). Early and late miscarriage rates and biochemical pregnancies showed no significant differences between the groups. **Conclusions:** Based on the results obtained, we could speculate that the depth of infiltration during HEFI does not significantly influence reproductive outcomes in oocyte recipients without intrauterine pathology. However, these findings should be interpreted with caution, given the limitations of the study, including its non-randomized design and differences among groups, such as variations in the number of embryos transferred and intraoperative group allocation. Therefore, further randomized clinical trials should be performed to more comprehensively understand the impact of incision depth on reproductive outcomes.

## 1. Introduction

Hysteroscopy is regarded as the golden standard for the assessment of the uterine cavity, enabling the accurate diagnosis of numerous underlying endometrial pathologies [1]. Furthermore, endometrial injury, commonly referred in the literature as “endometrial scratching” has gained increasing attention in recent years because of its potential beneficial effect on implantation rates [2,3,4,5]. In accordance with the meta-analysis written by Papanikolaou et al., hysteroscopy combined with endometrial scratching prior to embryo transfer was associated with a statistically significant improvement in clinical pregnancy and live birth rates, without an increased risk of miscarriage [6].

For a subset of patients classified as poor implanters, characterized by multiple failed IVF attempts using autologous oocytes, oocyte donation is commonly offered as a therapeutic option [7,8]. Even though oocyte donation is considered as one of the most effective assisted reproduction methods because oocytes donors are young and have no significant medical history, delivery rates remain only at around 50%, strongly suggesting that additional maternal factors could contribute to reproductive outcomes [8]. This cohort of women represents an ideal study population for investigations focusing on endometrial scratching, as it minimizes confounding variables related to the embryo quality and its developmental stage. Given that oocyte donors are typically young and of proven fertility, the resulting data may be considered more robust, allowing for more an accurate assessment of the isolated effect of endometrial injury on implantation and pregnancy rates in assisted reproductive technology (ART) cycles [9].

Hysteroscopic endometrial fundal incision (HEFI) is a unique endometrial scratching technique performed during mini-hysteroscopy. Several manuscripts demonstrate the potential clinical value of HEFI for improving endometrial receptivity and supporting implantation outcomes in assisted reproductive technology (ART) settings in oocyte recipients [10,11,12]. The endometrial scratching (incision) technique using endoscopic cold scissors enables a targeted and fully controlled endometrial incision performed in real time at the uterine fundus, which is one of the most common sites of embryo implantation [13].

However, during research and the implementation of the HEFI method, it was found that the thickness of the endometrial tissue and the underlying stromal layers above the myometrium of the uterine cavity could vary significantly among patients. Therefore, the actual depth of the incision required to reach the layer of first intra-endometrial vascular structures varied significantly, ranging from 2 mm to more than 8 mm.

Till now, no studies have yet evaluated the influence of the depth of controlled endometrial injury on reproductive outcomes in oocyte recipients undergoing assisted reproductive technology. Findings from such experiments are pivotal not only for further understanding the complex mechanisms of embryo implantation but, more importantly, for their potential clinical relevance, as they might contribute to the development of novel strategies aimed at improving reproductive outcomes [5].

The primary objective of this study was to evaluate the potential relationship between the depth of endometrial incision (HEFI) and reproductive outcomes in oocyte recipients undergoing IVF treatment. Specifically, we aimed to determine whether different incision depths, classified as superficial, intermediate, and deep, were associated with variations in clinical pregnancy rate, miscarriage risk, and live birth rate.

## 2. Materials and Methods

### 2.1. Description of the Study Group

A prospective study was conducted in patients with primary or secondary infertility of various etiologies, who underwent mini-hysteroscopy with endometrial fundal incision (HEFI), followed by frozen embryo transfer within two months of the procedure as part of infertility management in a cohort of oocyte recipients at the Assisting Nature Center for Reproduction and Genetics, Thessaloniki, Greece. Patients were carefully selected based on strict inclusion and exclusion criteria. The study group was designed to ensure maximum homogeneity. The study period extended from 1 November 2020 to 31 December 2024.

All participants provided written informed consent before enrollment. The trial was registered on ClinicalTrials.gov (NCT04580056) on 2 October 2020, and the study protocol received approval from the Institutional Review Board of the Assisting Nature In Vitro Fertilization Unit (Registration Number: 0210201405, Approval Date: 2 October 2020).

Inclusion criteria for patients undergoing hysteroscopy prior to oocyte donation included age between 25 and 50 years old, planned frozen blastocyst transfer only, and the absence of significant endometrial pathology such as submucosal fibroids or polyps and classification to the ≤U2 group by ESGE as confirmed by two- and three-dimensional ultrasonography, hysterosalpingography, or office hysteroscopy.

Exclusion criteria were: office hysteroscopy within six months before oocyte treatment, severe male factor infertility defined as sperm count <15 million/mL, total motility <40%, or normal forms <4% as per the World Health Organization criteria, history of uterine surgery, free fluid in the endometrial cavity during HRT preparation, unilateral or bilateral hydrosalpinx, severe adenomyosis, submucous uterine leiomyomas (FIGO 0–2), endometrial polyps, Müllerian malformations, and body mass index (BMI) greater than 35 kg/m^2^ due to decreased implantation rates. These criteria are consistent with the strategies aimed at optimizing uterine and endometrial conditions prior to embryo transfer and excluding factors known to adversely impact implantation and pregnancy outcomes, as supported by the American Society for Reproductive Medicine and the existing literature on IVF and donor oocyte cycles [14,15,16].

As a result, a total of 212 women without intrauterine pathology were included in the study group. Group allocation was based on intraoperative judgment. A detailed description and demographics of the participants are presented in Table 1.

Although statistically significant differences were observed in variables such as peak endometrial thickness, the absolute differences between groups were minimal. These findings, while possibly reflecting subtle trends, are unlikely to be of clinical relevance and should be interpreted with caution.

### 2.2. Hysteroscopic Endometrial Fundal Incision (HEFI) Classification

**Figure 1:**
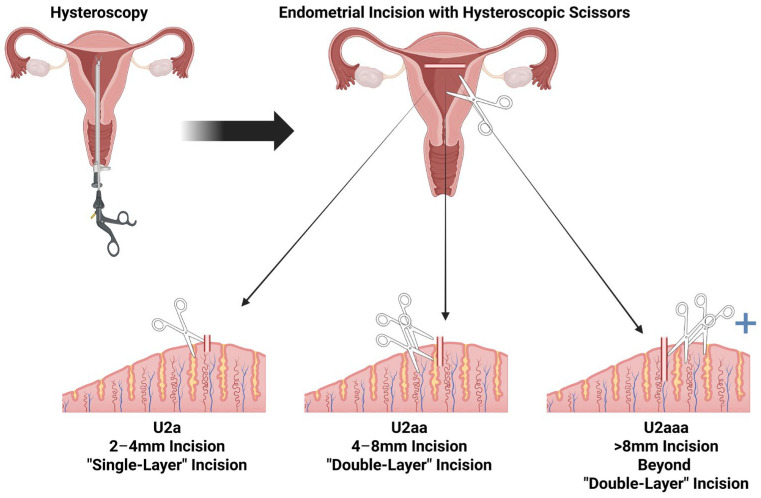
Classification of endometrial incision depth. A graphic representation of the classification. As showed by the bold arrow after visualization, during hysteroscopy three depth of incision could be performed presented by three thin arrows with red lines representing the incision, a single scissor incision referring to a depth of a single length of hysteroscopic scissors, a double scissor length or more than a double scissor length (+sign).

A protocol of the study assumed that all patients, after performing HEFI, would be classified according to the depth of endometrial incision into three groups. Table 2 illustrates the proposed three-level classification of endometrial incision depth observed during hysteroscopic procedures. The classification is based on the progressive depth of tissue penetration, defined by the visibility of vascular structures and the relative thickness of the incision. This standardized terminology (U2a, U2aa, U2aaa) allows for more precise description, reproducibility, and comparison of results across clinical studies (Figure 1).

### 2.3. Hysteroscopic Procedure Description

To optimize endometrial conditions and enhance visualization of the uterine cavity, patients received combined oral contraceptives prior to the procedure. This regimen was administered for one to two cycles before the initiation of endometrial preparation. Ovarian suppression achieved with oral contraceptives resulted in adequate endometrial thinning, providing optimal conditions for uterine cavity assessment and intervention prior to hormone replacement therapy (HRT) [17].

The hysteroscopic procedure was scheduled one to two months prior to the initiation of hormonal replacement therapy for endometrial preparation. To enhance visualization of the uterine cavity, patients began a combined oral contraceptive pill on day 3 of the cycle. The hysteroscopy was performed between days 6 and 13 during oral contraception cycle. A vaginoscopic approach was implemented, which omits the use of a speculum or tenaculum and is associated with reduced procedural pain and similar efficacy in comparison to traditional techniques [18]. Pain management was achieved using standard analgesics, with or without mild sedation, depending on the patient’s tolerance [19].

A rigid hysteroscope (4.8 mm, 30° forward oblique) was used, with 0.9% normal saline as the distension medium, consistent with best practices for visualization and patient’s safety [20]. Systematic inspection of the uterine cavity was performed after adequate distension, as recommended in scientific manuscripts and clinical practice recommendations [21,22,23]. All hysteroscopies were performed by two highly experienced surgeons using a single, standardized protocol.

HEFI was performed exclusively with endoscopic scissors with no electrocautery. Endometrial fundal incisions were made using a 2 mm endoscopic scissor, with the incision extending from one tubal ostium to the other in a straight line. The depth of the incision was determined by the appearance of the first visible vessels in the connective tissue and categorized by the surgeon’s intraoperative judgment as U2a—superficial, U2aa—intermediate, or U2aaa—deep, respectively, based on the depth of instrument penetration into the tissue, with one incision “layer” corresponding to the length of the endoscopic scissors.

### 2.4. Hormone Replacement Treatment Protocol

For endometrial preparation in frozen embryo transfer cycles using donor oocytes, oral 17-beta estradiol was initiated on cycle day 2 (baseline day), provided that transvaginal ultrasound confirmed quiescent ovaries, and baseline hormone levels were within the normal range. Estradiol was administered in a stepwise escalating regimen until the pregnancy test. Estrogen was administered for a minimum of 10 days and up to 20 days, depending on ultrasound-assessed endometrial response [24,25].

Between days 10 and 11, serum progesterone, luteinizing hormone, and estradiol were measured, and endometrial thickness was assessed with ultrasound. If the endometrial thickness was less than 7 mm, estrogen therapy was extended for three additional days. Once the endometrium exceeded 7 mm, daily progesterone was started, and embryo transfer was scheduled six days later. All transferred embryos were day 5 blastocysts. Serum beta-hCG was measured nine days after embryo transfer or 14 days after starting progesterone.

### 2.5. Assessment of the Reproductive Outcomes

Primary outcomes included positive pregnancy rate and live birth rate. Secondary outcomes included biochemical pregnancy, clinical pregnancy, and miscarriage rates.

Positive pregnancy was defined as a serum β-hCG level of >10 mIU/mL following embryo transfer. Live birth rate was defined as delivery of a live infant beyond 24 weeks of gestation [26,27].

Secondary outcomes included biochemical pregnancy, clinical pregnancy, and miscarriage rates. Clinical pregnancy was confirmed by the presence of fetal cardiac activity on ultrasound at 6–7 weeks of gestation. Biochemical pregnancy referred to a transient elevation in β-hCG without ultrasound evidence of a gestational sac. Miscarriages were categorized as early (<12 weeks) or late (12–24 weeks of gestation) [26,27,28,29,30].

### 2.6. Statistical Analysis

Chi-square and Fisher’s exact tests [31] were used to analyze categorical variables, while independent-samples *t*-tests were applied to continuous variables [32]. Statistical significance was defined as *p* < 0.05. A multivariate logistic regression analysis was performed to evaluate factors associated with live birth. The dependent variable was live birth. Independent variables included the depth of endometrial incision (U2a, U2aa, U2aaa), number of embryos transferred, endometrial thickness, and donor age. Additional covariates, such as number of previous embryo transfers and embryo stage, were included where available. Odds ratios (ORs) with 95% confidence intervals (CIs) were calculated. All statistical analyses were performed using SPSS version 25.0.

### 2.7. Ethical Standards of the Study

All participants provided written informed consent before enrollment. The trial was registered on ClinicalTrials.gov (NCT04580056) on 2 October 2020, and the study protocol received formal approval from the Institutional Review Board of the Assisting Nature In Vitro Fertilization Unit (Registration Number: 0210201405, Approval Date: 2 October 2020). No financial compensation was offered for the participants. All hysteroscopic procedures were performed with no additional costs for the participants. This conduct is consistent with ethical standards for clinical research in assisted reproduction, as described in the medical literature, which emphasizes the necessity of informed consent, prospective trial registration, institutional review board approval, and avoidance of undue inducement or financial burden for participants [33,34].

The present study was conducted within a broader prospective research program that originated in 2018 and evolved over time as additional research objectives and variables were included. The study was registered at ClinicalTrials.gov (NCT04580056), with registration completed prior to the initiation of data collection for the current analysis.

For the analyses presented in this manuscript, patient recruitment and data collection were conducted between 1 November 2020 and 31 December 2024. The specific variables examined in the current study, including the assessment of incision depth and tissue disruption, were prospectively collected only from 1 November 2020 onwards. Consequently, no patient data collected before 1 November 2020 were included in the presented analyses.

Although the broader research program generated previous publications, those studies were conducted independently, addressed different research questions, and utilized diffent datasets. There is no overlap between the patient data analyzed in the current manuscript and the data reported in earlier publications.

## 3. Results

The comparison of reproductive outcomes among study groups is presented in Table 3.

**Figure 2:**
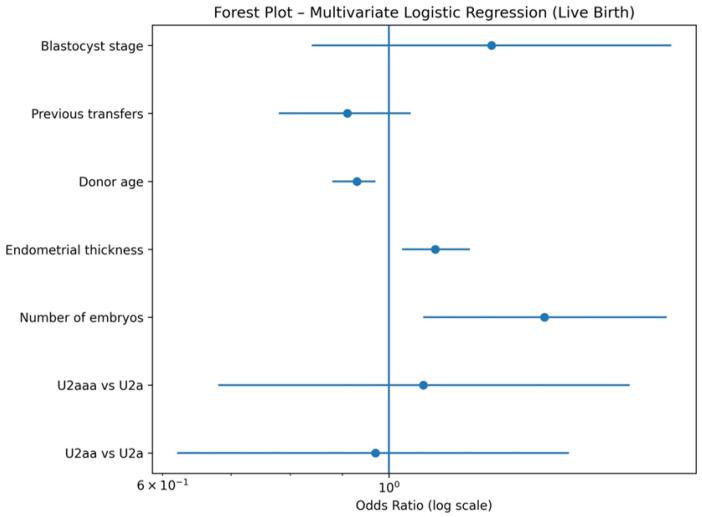
Forest plot of multivariate logistic regression analysis for live birth. Legend: ● represents the odds ratio (OR), horizontal lines indicate 95% confidence intervals (CI), and the vertical line at OR = 1 denotes no effect.

There were no statistically significant differences in positive β-hCG rates (*p* = 0.95), biochemical pregnancies (*p* = 0.95), clinical pregnancies (*p* = 0.96), or live birth rates (*p* = 0.97) among the groups (Table 4, Figure 2).

Likewise, early (<12 weeks) and late (12–24 weeks) miscarriage rates did not differ significantly, with *p* = 0.79 and *p* = 0.61, respectively. These results suggest that reproductive outcomes were comparable across the three studied subgroups.

No collinearity was observed between variables included in the model. Importantly, after adjustment for key clinical variables, the depth of incision remained non-significant, confirming that the observed outcomes were not confounded by differences in patient or treatment characteristics. The analysis demonstrates that the depth of endometrial incision (U2aa and U2aaa vs. U2a) was not significantly associated with live birth. In contrast, endometrial thickness and number of embryos transferred were significant positive predictors, while donor age showed a negative association.

Odds ratios (ORs) with 95% confidence intervals (CIs) are presented for each outcome, using the U2a group as the reference category. Crude ORs were calculated from univariate analyses, while adjusted ORs were derived from multivariate logistic regression models. The adjusted models included donor age, number of embryos transferred, endometrial thickness, and number of previous embryo transfers as covariates. An OR > 1 indicates a higher likelihood of the outcome compared to the reference group, whereas an OR < 1 indicates a lower likelihood. Statistical significance was defined as *p* < 0.05.

No statistically significant associations were observed between incision depth and reproductive outcomes, as all confidence intervals crossed unity. Both crude and adjusted analyses demonstrated no significant differences in reproductive outcomes across the three study groups. In the multivariate model, the depth of endometrial incision was not independently associated with live birth (U2aa: adjusted OR 0.97, 95% CI 0.52–1.80; U2aaa: adjusted OR 1.05, 95% CI 0.54–2.05). Similarly, no significant associations were observed for clinical pregnancy or positive β-hCG rates (Table 5).

## 4. Discussion

The analysis performed demonstrated that varying depths of infiltration during hysteroscopic endometrial fundal incision (HEFI), classified as U2a (superficial), U2aa (intermediate), and U2aaa (deep) which were performed accordingly to the anatomical and individual characteristics of each patient while consistently adhering to the protocol involving penetration of the myometrial layer of the uterine fundus, did not result in significant alterations in the pregnancy, clinical pregnancy, miscarriage, or live birth rates.

Our previous randomized controlled trial in oocyte recipients showed that endometrial fundal incision (HEFI) improved pregnancy rates compared with diagnostic hysteroscopy alone, which could suggest that endometrial injury could enhance implantation potential [10].

Endometrial scratching has been proposed to exert potential beneficial effects on implantation through several biological mechanisms, including: promotion of decidualization; upregulation of cytokines and growth factors, such as heparin-binding epidermal growth factor (*HB-EGF*), leukemia inhibitory growth factor (*LIF)*, and interleukin-11 (*IL-11*), as well as the recruitment and activation of immune cells, including macrophages and dendritic cells, and potential improvement in synchronization between endometrial receptivity and embryo development following endometrial injury [35,36,37].

In line with this, a systematic review and meta-analysis reported that intentional endometrial injury may increase clinical pregnancy rates in selected populations, particularly for those with repeated implantation failure (RIF). However, subgroup analyses demonstrated no consistent benefit for live birth or clinical pregnancy rates after adjustment for confounding factors, including maternal age, the number of previous failed cycles, and the method or timing of the injury [34,38].

These findings suggest the hypothesis of a threshold effect, whereby, once an adequate degree of endometrial injury is achieved, the endometrial response might become relatively uniform across patients, and a favorable inflammatory response and decidualization cascade could be initiated. In practical terms, this threshold has been proposed to correspond to an injury extending beyond the superficial endometrial layers, potentially involving the myometrial interface and initial vascular structures. Collectively, these mechanisms might contribute to improved endometrial gene expression and enhance endometrial receptivity [39,40].

Based on acquired findings, several important conclusions could be suggested:

First: The protocol and methodology of our mini-hysteroscopic technique should be consistent and appropriate. The decision to incise the endometrium until the first appearance of sub-endometrial vessels could be justified and correct in light of the final reproductive outcomes observed after the application of this method.

Second: In this large cohort of patients, we observed subtle anatomical variations in the structure of the incised uterine wall that are not currently addressed by the ESGE/ESHRE classification systems [41]. Specifically, these variations involved the variable thickness of connective tissue between the endometrium, which undergoes cyclic regeneration and lines the uterine cavity and the underlying myometrium, where the vascular network is located. This observation is particularly noteworthy, as it is consistent with recent reports in the literature [42,43]. Based on these clinical observations, such cases were interpreted within the spectrum of minimal uterine septal variants and were conceptually aligned with group U2 in our proposed classification framework. This approach may provide further insights into the potential impact of subtle anatomical variations on endometrial receptivity and implantation capacity.

Third: Collectively, these findings could further suggest the safety and efficacy of HEFI in oocyte recipients’ treatments.

By focusing exclusively on oocyte recipients, thereby controlling for embryo quality and minimizing age-related endometrial variability, the presented study allows a more focused assessment of the direct uterine response to HEFI. Our previous investigations in oocyte donation cycles have similarly demonstrated significant improvements in clinical pregnancy and live birth rates with hysteroscopically controlled local endometrial injury [7,11]. Additionally, both meta-analyses and randomized controlled trials highlighted substantial heterogeneity in studies’ methodology, timing of intervention, and patient selection, which complicates the interpretation of the overall efficacy of endometrial injury [7,38,44].

Importantly, deeper incisions did not increase adverse outcomes such as miscarriage, which could suggest the procedural flexibility of selecting incision depth according to intraoperative visualization of fundal vessels without compromising efficacy [11,45]. The safety of hysteroscopic procedures, when performed with standardized protocols, is well established in the literature [45].

Although minor differences were observed in peak endometrial thickness across the groups, these variations did not translate into clinically meaningful differences in reproductive outcomes. While such parameters were considered potential confounders, their minimal variability could further suggest the robustness of the HEFI technique, irrespective of slight demographic or endometrial shifts [7,11,38,46].

Finally, several limitations of the study should be discussed. First of all, patient allocation to groups was non-randomized and determined post hoc based on hysteroscopic findings. Consequently, the number of patients, embryos, and transfers within each group was not predefined but rather reflected the observed depth of endometrial incision. We acknowledge that this may represent a potential limitation of the study, as it does not allow for the complete elimination of possible statistical bias. Additionally, the number of transferred embryos was not standardized as one or two embryos were transferred, depending primarily on embryo availability and patient preference; this variation represents a potential source of imbalance and may act as a confounding factor influencing the final study outcomes. Moreover, even though surgeries were performed by two experienced surgeons in accordance with a single standardized protocol, an intra- and inter-observer variability could have happened among the cases. Finally, the classification was based on operators’ intraoperative visual assessment of the endometrium, as no further imaging validation could be applied.

## 5. Conclusions

HEFI could represent a standardized and reproducible approach aimed at enhancing endometrial receptivity in assisted reproduction. Despite classification into superficial (U2a), intermediate (U2aa), and deep (U2aaa) injury groups, and adjustment for anatomical and individual patient characteristics, no significant differences were observed in pregnancy, clinical pregnancy, miscarriage, or live birth rates. Our findings support the hypothesis of a threshold effect: whereby, once an adequate depth of endometrial injury is achieved, the subsequent endometrial response appears to be relatively uniform across patients. This might underscore that the key determinant of outcome is adherence to a standardized protocol, while allowing for individual anatomical variations in the uterine wall. Although previous meta-analyses showed heterogeneous results across different patient populations, the exclusive inclusion of oocyte recipients in the present study aimed to minimize major confounding factors such as embryo quality and maternal age. Minor differences in endometrial thickness were not associated with clinically meaningful outcome differences, which could strengthen the reproducibility of this approach. While results acquired through this study should be interpreted with caution, HEFI appears to be a promising strategy for modulating endometrial receptivity in assisted reproductive technology. However, prospective randomized studies should be performed in order to optimize procedural parameters and to confirm these findings across broader and more diverse patient populations.

## Figures and Tables

**Table 1:** Characteristics of the study group.

	U2a (n = 82)	U2aa (n = 91)	U2aaa (n = 39)	*p*-Value
Age (years ± SD)	40.2 (±6.8)	39.4 (±5.5)	40.4 (±7.1)	0.60
BMI (kg/m^2^)	27.3 (±5.1)	26.1 (±4.1)	27.6 (±6.8)	0.47
Duration of infertility (years)	9.1 (±3.9)	8.5 (±4.4)	10 (±6.2)	0.91
Donor’s age, mean (SD)	26.3 (+4.5)	27.1 (+5.1)	25.9 (+4.4)	0.01
Mean number of blastocysts transferred	1.6 (±0.4)	1.7 (±0.4)	1.3 (±0.3)	<0.001
Mean total number of blastocysts available for transfer	4.5 (±1.8)	4.6 (±2.0)	4.1 (±1.6)	0.015
Peak endometrial thickness (mm)	10.1 (±1.2)	10.2 (±1.1)	9.7 (±1)	0.008
Endometrial preparation duration (days), mean (SD)	17.4 (±3.6)	16.8 (±4.1)	17.1 (±3.9)	0.33
Previous live birth, n (%)	8 (9.8%)	11 (12%)	1 (2.6%).	0.23
Previous miscarriage, n (%)	3 (3.65%)	5 (6%)	2 (5.2%)	0.84
Previous biochemical pregnancy, n (%)	9 (10.9%)	11 (13.4%)	7 (8.54%)	0.54
Previous ectopic pregnancy, n (%)	1 (1.2%)	0 (0%)	0	0.45
Previously induced abortion, n (%)	2 (2.4%)	1 (1.7%)	0	0.53

EFI: endometrial fundal incision, BMI: body mass index, HRT: hormone replacement treatment.

**Table 2:** Classification of endometrial incision depth according to hysteroscopic technique HEFI.

Depth of Incision	Definition	Classification
2–4 mm	Single-layer incision, corresponding to the width of hysteroscopic scissors	Superficial level (U2a)
4–8 mm	Double-layer incision	Intermediate level (U2aa)
>8 mm	Incision beyond double-layer depth	Deepest level (U2aaa)

**Table 3:** Reproductive outcomes in the study population.

Outcome	U2a (n = 82)	U2aa (n = 91)	U2aaa (n = 39)	*p*-Value
Positive b-hCG	78% (n = 64)	76.9% (n = 70)	77.1% (n = 31)	0.95
Biochemical pregnancy	6.1% (n = 5)	6.6% (n = 6)	7.6% (n = 3)	0.95
Early miscarriage rate (<12 weeks)	12.2% (n = 10)	11% (n = 10)	15.3% (n = 6)	0.79
Late miscarriage rate (12–24 weeks)	1.2% (n = 1)	2.2% (n = 2)	0% (n = 0)	0.61
Clinical pregnancy rate	69.5% (n = 57)	69.2% (n = 63)	71.8% (n = 28)	0.96
Live birth rate	58.5% (n = 48)	57.1% (n = 52)	56.4% (n = 22)	0.97

**Table 4:** Multivariate logistic regression analysis for live birth outcome.

Variable	OR	95% CI	*p*-Value
Depth of incision			
U2a (reference)	1.00	–	–
U2aa	0.97	0.62–1.50	0.89
U2aaa	1.08	0.68–1.72	0.74
Number of embryos transferred	1.42	1.08–1.87	0.01
Endometrial thickness (mm)	1.11	1.03–1.20	0.006
Donor age (years)	0.93	0.88–0.97	0.002
Previous embryo transfers	0.91	0.78–1.05	0.18
Blastocyst stage transfer	1.26	0.84–1.89	0.26

**Table 5:** Crude and adjusted odds ratios for reproductive outcomes. U2Aa has been chosen as a reference.

Outcome	Group	Crude OR (95% CI)	Adjusted OR (95% CI) *	*p*-Value
Live birth	U2a	1.00 (reference)	1.00 (reference)	–
	U2aa	0.94 (0.51–1.73)	0.97 (0.52–1.80)	0.91
	U2aaa	0.92 (0.43–1.98)	1.05 (0.54–2.05)	0.88
Clinical pregnancy	U2a	1.00	1.00	–
	U2aa	0.98 (0.52–1.84)	1.01 (0.53–1.92)	0.97
	U2aaa	1.11 (0.49–2.53)	1.15 (0.50–2.65)	0.74
Positive β-hCG	U2a	1.00	1.00	–
	U2aa	0.93 (0.48–1.80)	0.95 (0.49–1.86)	0.88
	U2aaa	0.96 (0.38–2.41)	1.02 (0.40–2.60)	0.96

* Calculated from multivariate logistic regression model.

## Data Availability

All relevant data are included within the manuscript. The raw data are available on request from the corresponding author.

## Abbreviations

The following abbreviations are used in this manuscript:

IVF	In Vitro Fertilization
HEFI	Hysteroscopic Endometrial Fundal Incision
ART	Assisted Reproductive Technology
BMI	Body Mass Index
HRT	Hormone Replacement Therapy
ESGE	European Society for Gynaecological Endoscopy
β-hCG	Beta-Human Chorionic Gonadotropin
*HB-EGF*	Heparin-Binding Epidermal Growth Factor
LIF	Leukemia Inhibitory Growth Factor
IL 11	Interleukin-11

## References

[1] Riemma G., Vitale S.G., Manchanda R., Rathore A., Torok P., De Angelis C., Urman B., Iraci Sareri M., La Verde M., Carugno J. (2022). The role of hysteroscopy in reproductive surgery: Today and tomorrow. J. Gynecol. Obstet. Hum. Reprod..

[2] Zygula A., Szymusik I., Grzechocinska B., Marianowski P., Wielgos M. (2016). Endometrial injury for women with previous in vitro fertilization failure—Does it improve pregnancy rate?. Neuro Endocrinol. Lett..

[3] Siristatidis C., Kreatsa M., Koutlaki N., Galazios G., Pergialiotis V., Papantoniou N. (2017). Endometrial injury for RIF patients undergoing IVF/ICSI: A prospective nonrandomized controlled trial. Gynecol. Endocrinol..

[4] Olesen M.S., Hauge B., Ohrt L., Olesen T.N., Roskaer J., Baek V., Elbaek H.O., Nohr B., Nyegaard M., Overgaard M.T. (2019). Therapeutic endometrial scratching and implantation after in vitro fertilization: A multicenter randomized controlled trial. Fertil. Steril..

[5] Sar-Shalom Nahshon C., Sagi-Dain L., Wiener-Megnazi Z., Dirnfeld M. (2019). The impact of intentional endometrial injury on reproductive outcomes: A systematic review and meta-analysis. Hum. Reprod. Update.

[6] Papanikolaou E., Peitsidis N., Tsakiridis I., Michos G., Skalias A., Patoulias D., Poutoglidis A., Mamopoulos A., Athanasiadis A., Grimpizis G. (2023). Endometrial scratching during hysteroscopy in women undergoing in vitro fertilization: A systematic review and meta-analysis. Front. Surg..

[7] Dain L., Ojha K., Bider D., Levron J., Zinchenko V., Walster S., Dirnfeld M. (2014). Effect of local endometrial injury on pregnancy outcomes in ovum donation cycles. Fertil. Steril..

[8] Coughlan C. (2018). What to do when good-quality embryos repeatedly fail to implant. Best. Pract. Res. Clin. Obstet. Gynaecol..

[9] Lacconi V., Massimiani M., Carriero I., Bianco C., Ticconi C., Pavone V., Alteri A., Muzii L., Rago R., Pisaturo V. (2024). When the Embryo Meets the Endometrium: Identifying the Features Required for Successful Embryo Implantation. Int. J. Mol. Sci..

[10] Peitsidis N., Tsakiridis I., Najdecki R., Michos G., Chouliara F., Timotheou E., Chartomatsidou T., Athanasiadis A., Papanikolaou E. (2023). Diagnostic hysteroscopy with endometrial fundal incision may improve reproductive outcomes in oocyte recipients after implantation failure. JBRA Assist. Reprod..

[11] Peitsidis N., Tsakiridis I., Najdecki R., Michos G., Kalogiannidis I., Athanasiadis A., Papanikolaou E. (2024). Hysteroscopic Endometrial Fundal Incision versus Hysteroscopy Only in Oocyte Recipients: A Randomized Controlled Trial Assessing The Reproductive Outcomes. Int. J. Fertil. Steril..

[12] Najdecki R., Peitsidis N., Tsakiridis I., Michos G., Timotheou E., Chartomatsidou T., Athanasiadis A., Papanikolaou E. (2023). Hysteroscopic Endometrial Fundal Incision in Oocyte Recipients before Embryo Transfer May Improve Reproductive Outcomes: A Prospective Study. Int. J. Fertil. Steril..

[13] Minami S., Ishihara K., Araki T. (2003). Determination of blastocyst implantation site in spontaneous pregnancies using three-dimensional transvaginal ultrasound. J. Nippon. Med. Sch..

[14] Santoro N., Polotsky A.J. (2025). Infertility Evaluation and Treatment. N. Engl. J. Med..

[15] Practice Committee of the American Society for Reproductive Medicine (2021). Evidence-based outcomes after oocyte cryopreservation for donor oocyte in vitro fertilization and planned oocyte cryopreservation: A guideline. Fertil. Steril..

[16] Shi Y., Sun Y., Hao C., Zhang H., Wei D., Zhang Y., Zhu Y., Deng X., Qi X., Li H. (2018). Transfer of Fresh versus Frozen Embryos in Ovulatory Women. N. Engl. J. Med..

[17] Jeong N., Cho A., Koo Y.J., Ahn J.W., Park H., Lee E.S., Yi S.W., Joo W.D., Lee S.H., Lee J.K. (2025). Clinical practice in office hysteroscopy. Obstet. Gynecol. Sci..

[18] Turktekin N., Karakus C., Ozyurt R. (2022). Comparing the effects of endometrial injury with hysteroscopy or Pipelle cannula on fertility outcome. Eur. Rev. Med. Pharmacol. Sci..

[19] Khoiwal K., Zaman R., Bahurupi Y., Gaurav A., Chaturvedi J. (2024). Comparison of vaginoscopic hysteroscopy and traditional hysteroscopy: A systematic review and meta-analysis. Int. J. Gynaecol. Obstet..

[20] American College of Obstetricians and Gynecologists (2018). The American College of Obstetricians and Gynecologists: Women’s Health Care Physicians. Obstet. Gynecol..

[21] Smit J.G., Kasius J.C., Eijkemans M.J.C., Koks C.A.M., van Golde R., Nap A.W., Scheffer G.J., Manger P.A.P., Hoek A., Schoot B.C. (2016). Hysteroscopy before in-vitro fertilisation (inSIGHT): A multicentre, randomised controlled trial. Lancet.

[22] El-Toukhy T., Campo R., Khalaf Y., Tabanelli C., Gianaroli L., Gordts S.S., Gordts S., Mestdagh G., Mardesic T., Voboril J. (2016). Hysteroscopy in recurrent in-vitro fertilisation failure (TROPHY): A multicentre, randomised controlled trial. Lancet.

[23] (2020). The Use of Hysteroscopy for the Diagnosis and Treatment of Intrauterine Pathology: ACOG Committee Opinion, Number 800. Obstet. Gynecol..

[24] Practice Committee of the American Society for Reproductive Medicine (2024). The use of hormonal contraceptives in fertility treatments: A committee opinion. Fertil. Steril..

[25] Glujovsky D., Pesce R., Sueldo C., Quinteiro Retamar A.M., Hart R.J., Ciapponi A. (2020). Endometrial preparation for women undergoing embryo transfer with frozen embryos or embryos derived from donor oocytes. Cochrane Database Syst. Rev..

[26] Feng Q., Li W., Li W., Wang R., Crispin J., Longobardi S., D’Hooghe T., Mol B.W. (2025). The presence, clarity, and consistency of definitions in pregnancy outcomes in infertility trials: A systematic review. Hum. Reprod..

[27] Munoz E., Taboas E., Alvarez M., Gil E., Perez A., Portela S., Martinez-Chapela M., Saucedo E., Garrido N. (2024). Is biochemical pregnancy loss associated with embryo or endometrium? A retrospective cohort study in frozen single embryo transfer of own and donated oocytes. Hum. Reprod..

[28] Smith A., Tilling K., Nelson S.M., Lawlor D.A. (2015). Live-Birth Rate Associated with Repeat In Vitro Fertilization Treatment Cycles. JAMA.

[29] Vitagliano A., Di Spiezio Sardo A., Saccone G., Valenti G., Sapia F., Kamath M.S., Blaganje M., Andrisani A., Ambrosini G. (2018). Endometrial scratch injury for women with one or more previous failed embryo transfers: A systematic review and meta-analysis of randomized controlled trials. Fertil. Steril..

[30] Quenby S., Gallos I.D., Dhillon-Smith R.K., Podesek M., Stephenson M.D., Fisher J., Brosens J.J., Brewin J., Ramhorst R., Lucas E.S. (2021). Miscarriage matters: The epidemiological, physical, psychological, and economic costs of early pregnancy loss. Lancet.

[31] Kim H.Y. (2017). Statistical notes for clinical researchers: Chi-squared test and Fisher’s exact test. Restor. Dent. Endod..

[32] Mishra P., Pandey C.M., Singh U., Keshri A., Sabaretnam M. (2019). Selection of appropriate statistical methods for data analysis. Ann. Card. Anaesth..

[33] Lensen S., Osavlyuk D., Armstrong S., Stadelmann C., Hennes A., Napier E., Wilkinson J., Sadler L., Gupta D., Strandell A. (2019). A Randomized Trial of Endometrial Scratching before In Vitro Fertilization. N. Engl. J. Med..

[34] Lensen S.F., Armstrong S., Gibreel A., Nastri C.O., Raine-Fenning N., Martins W.P. (2021). Endometrial injury in women undergoing in vitro fertilisation (IVF). Cochrane Database Syst. Rev..

[35] Chen T., Shi H., Fang L.L., Su Y.C. (2020). The effect of endometrial injury on reproductive outcomes of frozen-thawed embryo transfer cycles in women with one implantation failure. J. Int. Med. Res..

[36] Lindekugel S.M.K., Frankel L.R., Deaton J.L., Dong A., Lassiter R., Savaris R.F., Lessey B.A. (2025). Endometrial scratching before euploid embryo transfer: A case-control study. J. Assist. Reprod. Genet..

[37] Izquierdo A., de la Fuente L., Spies K., Lora D., Galindo A. (2021). Is Endometrial Scratching Beneficial for Patients Undergoing a Donor-Egg Cycle with or without Previous Implantation Failures? Results of a Post-Hoc Analysis of an RCT. Diagnostics.

[38] Wang Y., Tang Z., Wang C., Teng X., He J. (2024). Whether hysteroscopy improves fertility outcomes in infertile women: A meta-analysis and systematic review. Front. Endocrinol..

[39] Ersahin S.S., Ersahin A. (2022). Endometrial injury concurrent with hysteroscopy increases the expression of Leukaemia inhibitory factor: A preliminary study. Reprod. Biol. Endocrinol..

[40] Pandur E., Pap R., Sipos K. (2024). Activated THP-1 Macrophage-Derived Factors Increase the Cytokine, Fractalkine, and EGF Secretions, the Invasion-Related MMP Production, and Antioxidant Activity of HEC-1A Endometrium Cells. Int. J. Mol. Sci..

[41] Grimbizis G.F., Gordts S., Di Spiezio Sardo A., Brucker S., De Angelis C., Gergolet M., Li T.C., Tanos V., Brolmann H., Gianaroli L. (2013). The ESHRE/ESGE consensus on the classification of female genital tract congenital anomalies. Hum. Reprod..

[42] Rasmussen C.K., Hansen E.S., Dueholm M. (2019). Two- and three-dimensional ultrasonographic features related to histopathology of the uterine endometrial-myometrial junctional zone. Acta Obstet. Gynecol. Scand..

[43] Chen F., Gong Y., Xie Y., Zhu L., Chen L., Xiao J., Jiang N., Sun L., Sui L. (2023). Assessment of key parameters of normal uterus in women of reproductive age. Sci. Rep..

[44] Gurgan T., Kalem Z., Kalem M.N., Ruso H., Benkhalifa M., Makrigiannakis A. (2019). Systematic and standardized hysteroscopic endometrial injury for treatment of recurrent implantation failure. Reprod. Biomed. Online.

[45] Munro M.G., Christianson L.A. (2015). Complications of Hysteroscopic and Uterine Resectoscopic Surgery. Clin. Obstet. Gynecol..

[46] Arce H., Velilla E., Lopez-Teijon M. (2016). Association between endometrial thickness in oocyte donation cycles and pregnancy success rates. Reprod. Fertil. Dev..

